# Tyrosol Facilitates Neovascularization by Enhancing Skeletal Muscle Cells Viability and Paracrine Function in Diabetic Hindlimb Ischemia Mice

**DOI:** 10.3389/fphar.2019.00909

**Published:** 2019-08-14

**Authors:** Jianqi Zhang, Dyah Ari Nugrahaningrum, Olivia Marcelina, Agnes Dwi Ariyanti, Guixue Wang, Caiping Liu, Shourong Wu, Vivi Kasim

**Affiliations:** ^1^The Key Laboratory of Biorheological Science and Technology, Ministry of Education, College of Bioengineering, Chongqing University, Chongqing, China; ^2^State and Local Joint Engineering Laboratory for Vascular Implants, Chongqing, China; ^3^The 111 Project Laboratory of Biomechanics and Tissue Repair, College of Bioengineering, Chongqing University, Chongqing, China

**Keywords:** tyrosol, diabetic hindlimb ischemia, skeletal muscle cells, cytoprotection, blood perfusion, therapeutic angiogenesis

## Abstract

As one of the most severe manifestations of diabetes, vascular complications are the main causes of diabetes-related morbidity and mortality. Hyperglycemia induces systemic abnormalities, including impaired angiogenesis, causing diabetic patients to be highly susceptible in suffering hindlimb ischemia (HLI). Despite its severe prognosis, there is currently no effective treatment for diabetic HLI. Skeletal muscle cells secrete multiple angiogenic factors, hence, recently are reported to be critical for angiogenesis; however, hyperglycemia disrupted the paracrine function in skeletal muscle cells, leading to the impaired angiogenesis potential observed in diabetic patients. The present study showed that tyrosol, a phenylethanoid compound, suppresses accumulation of intracellular reactive oxygen species (ROS) caused by hyperglycemia, most plausibly by promoting heme oxygenase-1 (HO-1) expression in skeletal muscle cells. Consequently, tyrosol exerts cytoprotective function against hyperglycemia-induced oxidative stress in skeletal muscle cells, increases their proliferation vigorously, and simultaneously suppresses apoptosis. Furthermore, tyrosol grossly increases the secretion of vascular endothelial growth factor-A (VEGF-A) and platelet-derived growth factor-BB (PDGF-BB) from skeletal muscle cells. This leads to enhanced proliferation and migration capabilities of vascular endothelial and smooth muscle cells, two types of cells that are responsible in forming blood vessels, through cell-cell communication. Finally, *in vivo* experiment using the diabetic HLI mouse model showed that tyrosol injection into the gastrocnemius muscle of the ischemic hindlimb significantly enhances the formation of functional blood vessels and subsequently leads to significant recovery of blood perfusion. Overall, our findings highlight the potential of the pharmacological application of tyrosol as a small molecule drug for therapeutic angiogenesis in diabetic HLI patients.

## Introduction

Diabetes mellitus is a common chronic metabolic disease that generates a great socioeconomic burden. It is accompanied by various complications, such as retinopathy, nephropathy, diabetic foot, and vascular diseases (Ramachandran et al., 2010; Rask-Madsen and King, 2013). As one of the most severe manifestations in diabetes, vascular complications are the main causes for diabetes-related morbidity and mortality (Beckman and Creager, 2016). Hindlimb ischemia (HLI) is one manifestation of peripheral artery disease (PAD) and occurs due to the obstruction of blood perfusion to the lower limb; herein, diabetic patients are faced with higher risk of HLI (Jude et al., 2010). Compared with non-diabetic patients, the lower-extremity amputation risks of diabetic patients are 10 times higher, and their mortality rate is more than three times higher, resulting in a significantly severe prognosis (Jude et al., 2010; Hoffstad et al., 2015). For correctly selected HLI patients, conventional treatments to promote revascularization in the lower limb, such as catheter- or stent-based surgery, could provide a >75% limb salvage rate within a year (Berceli et al., 2007). However, diabetic HLI patients show worse prognosis than non-diabetic ones, as indicated by higher recurrence rate and larger wound surface. Therefore, general treatments would result in more unfavorable outcomes (Lonborg et al., 2014).

Therapeutic angiogenesis is another promising strategy for HLI patients, including those who have been assumed as “no choice” for surgical-based therapy. It utilizes and improves innate physiological potential to promote neoangiogenesis and subsequently augment blood perfusion, which then compensates insufficient oxygen and nutrient supply to ischemic tissues (Annex, 2013). However, hyperglycemia induces systemic disruption of physiological functions, such as angiogenesis, by suppressing the expression of various crucial angiogenic factors, such as the vascular endothelial growth factor-A (VEGF-A) (Avogaro et al., 2011; Caporali et al., 2015) and the platelet-derived growth factor-BB (PDGF-BB) (Tanii et al., 2006). Therefore, effective induction of neoangiogenesis remains to be a problem for yielding a successful therapy in diabetic HLI patients.

As the largest paracrine and endocrine organ, skeletal muscle is known to exert predominant function by secreting numerous factors, including VEGF-A, PDGF-BB, fibroblast growth factor 2 (FGF2), and hepatocyte growth factor (HGF) (Perez-Ilzarbe et al., 2008; Giudice and Taylor, 2017). These factors positively influence vascular endothelial and smooth muscle cells, which are responsible for the formation of mature blood vessels, and subsequently enhance neoangiogenesis (Jain, 2003; Tateno et al., 2006; Perez-Ilzarbe et al., 2008). Our earlier results also showed that targeting and improving this paracrine function would be beneficial for the development of potential therapeutic strategy for HLI (Wu et al., 2015; Zhang et al., 2017). Despite its promising therapeutic outcome, the effective induction of skeletal muscle cell paracrine function in diabetic patients still poses a great challenge as hyperglycemia also alters the survival and secretory potentials of skeletal muscles (Giudice and Taylor, 2017; Teng and Huang, 2019).

Tyrosol [2-(4-hydroxyphenyl) ethanol] is a phenylethanoid natural compound present in various natural products, such as extra-virgin olive oil and white wine (St-Laurent-Thibault et al., 2011). It is also one major compound of *Rhodiola*, a high-altitude plant that has been exploited in traditional medicines (Gupta et al., 2008; Chiang et al., 2015; Torrens-Spence et al., 2018). In this study, we found that tyrosol induces the expression of the cytoprotective factor heme oxygenase-1 (HO-1) in skeletal muscle cells under hyperglycemia, which is crucial for oxidative stress protection (Iori et al., 2008; Gou et al., 2018), and concomitantly decreases hyperglycemia-induced accumulation of intracellular reactive oxygen species (ROS). It also enhances the viability of skeletal muscle cells by promoting their proliferation capability and simultaneously suppressing apoptosis. In addition, tyrosol restores hyperglycemia-induced disruption of the paracrine potential in skeletal muscle cells, leading to increased secretion of VEGF-A and PDGF-BB, which promotes the proliferation and migration capabilities of vascular endothelial and smooth muscle cells. Furthermore, intramuscular administration of tyrosol into the gastrocnemius muscle of diabetic HLI mice significantly enhances the formation of functional blood vessels, restoring blood perfusion in diabetic HLI mice. Thus, the present study provides a novel strategy for therapeutic angiogenesis in diabetic HLI using the small molecule drug tyrosol.

## Materials and Methods

### Cell Culture and Reagents

The skeletal muscle cell line C2C12, human umbilical vein endothelial cells (HUVECs), and smooth muscle cell line MOVAS were obtained from the American Type Culture Collection (ATCC). All cells were cultured in Dulbecco’s modified Eagle medium basic (Gibco, Life Technologies, Grand Island, NY) supplemented with 10% fetal bovine serum (FBS) (Biological Industries, Beit Haemek, Israel) in a humidified incubator (37°C, 5%CO_2_). Detection for mycoplasma contamination in all cell lines was done regularly using Mycoplasma Detection Kit-QuickTest (Biotool, Houston, TX), and these were found to be negative. Tyrosol was purchased from Sigma Aldrich (St. Louis, MO; molecular weight, 138; purity, ≥ 99.5%). Zinc protoporphyrin IX (ZnPP) was purchased from APExBIO (Houston, TX), whereas Ki8751 and CP868596 were purchased from MedChem Express (Monmouth Junction, NJ).

For induction of hyperglycemia condition, cells were re-seeded, and 24 h later, the cells were cultured in high-glucose medium (final concentration, 25 mM) (Kapitulnik et al., 2012; Dokun et al., 2014). Twenty-four hours later, cells were treated with tyrosol (final concentration, 50 mg/ml) or phosphate-buffered saline (PBS). After 24 h, cells were subjected into media without FBS prior to hypoxia exposure in a hypoxia chamber (Anaeropouch Box, 0.1% O_2_; Mitsubishi GAS Chemical, Tokyo, Japan) as previously described (Zhang et al., 2017). As the control, cells were cultured in 5.5 mM glucose and treated with PBS.

For experiments using HO-1 inhibitor, cells were treated with ZnPP (final concentration, 10 µM) for 12 h prior to treatment with tyrosol. For experiments using VEGFR and PDGFR-β inhibitors, cells were cultured with medium containing Ki8751 (final concentration, 2 nM) or CP868596 (final concentration, 0.3 µM), respectively, for 12 h prior to being cultured with conditioned media.

For HO-1 knockdown, cells were seeded in a six-well plate prior to transfection. Control vector (shCon) or HO-1-specific shRNA expression vectors (shHO-1) were transfected into the cells using Lipofectamine 2000 (Invitrogen Life Technologies, Grand Island, NY) according to the manufacturer’s instruction. Twenty-four hours later, selection for eliminating untransfected cells were performed using puromycin (2.5 mg/ml) for 36 h.

### Plasmids and Constructs

Murine HO-1 (NM_010442) shRNA expression vectors (shHO-1-1 and shHO-1-2) were constructed as described previously (Miyagishi and Taira, 2003), and the RNAi target sites are as follows: shHO1-1: GGGAGATACCTGACACAGT; shHO-1-2: GGGAAACCCCAGATCAGCA. For control vector (shCon), a vector containing a stretch of seven thymines downstream to the U6 promoter was used.

### Animal Experiment and Diabetic HLI Model

Male C57BL/6 mice (8 weeks old; body weight, 19-25 g) were acquired from the Third Military Medical University (Chongqing, China). All animal studies were performed in the Third Military Medical University, approved by the Laboratory Animal Welfare and Ethics Committee of the Third Military Medical University and handled according to the Guide for the Care and Use of Laboratory Animals of the Ministry of Health, China. Mice anesthesia was done by injecting solution containing ketamine (80 mg/kg)/xylazine (5 mg/kg) intraperitoneally.

Briefly, diabetic model was established by high-fat diet feeding to the mice (20% kcal protein, 20% kcal carbohydrate, and 60% kcal fat) for 3 weeks. At the same time, regular injection of streptozotocin (Sigma Aldrich; 40 mg/kg/day dissolved in sodium citrate buffer) was done intraperitoneally for 5 days in a row (Han et al., 2019). Accu-Check Integra (Roche Diagnostics; Shanghai, China) was used to check the blood glucose level at appointed time points, which are as follows: prior to streptozotocin injection, 1 week after the last injection, and at the end of the experiments. Mice were fasted overnight before blood glucose measurement. Diabetic mice model was considered successful with blood glucose level ≥16.7 mmol/L and further used for establishing diabetic HLI model (Scholz et al., 2002; Zhou et al., 2017). Prior to HLI model establishment, mice were anesthetized and the hindlimb area was depilated. Complete excision was performed on the proximal part of the left femoral artery, whereas the right one was left unexcised as a control. One day after the artery excision, tyrosol (50 mg/kg body weight dissolved in PBS) or PBS (control) was administered into the gastrocnemius muscle and every 3 days thereafter. Mice were grouped randomly after the surgery. The investigator was blinded during the assessment.

### Laser Doppler Perfusion Imaging

Laser Doppler Perfusion Imaging instrument (Moor Instruments Ltd, Axminster, Devon, England) was used to measure the blood perfusion in anesthetized mice at appointed time points. The ratio of blood perfusion was calculated by normalizing the blood perfusion in ischemic hindlimb (left) to the non-ischemic hindlimb (right) as reported previously (Tanii et al., 2006; Wu et al., 2015; Zhang et al., 2017).

### Preparation of Conditioned Medium

Conditioned medium was prepared by collecting the C2C12 culture medium and filtrating it through a 0.22-μm filter. Previously, C2C12 cells were treated with tyrosol (final concentration, 50 μg/ml) for 24 h, as previously described. Cells were then washed and exposed to hypoxia under hyperglycemia for another 24 h, followed by conditioned medium (CM-H/Tyr) collection. As a control, PBS was added instead of tyrosol for 24 h, followed by washing and exposure to hypoxia in normoglycemia or hyperglycemia (CM-C and CM-H, respectively).

### Enzyme-Linked Immunosorbent Assay (ELISA)

After collecting the conditioned media, the amount of VEGF-A in the conditioned media was analyzed using Mouse VEGF ELISA kit (Neobioscience, Shenzhen, China), and the amount of PDGF-BB was analyzed using Mouse PDGF-BB ELISA Kit (Yuanye, Shanghai, China) following the manufacturer’s guidelines.

### EdU Incorporation Assay

Prior to 5-Ethynyl-2'-deoxyuridine (EdU) incorporation assay, cells were treated with tyrosol (final concentration, 50 μg/ml) or PBS (as a control) prior to hypoxia exposure, as previously described. EdU incorporation and staining was then performed using Cell-Light EdU Apollo 488 *In Vitro* Imaging Kit (RiboBio, Guangzhou, China). Nuclei were stained with Hoechst, and procedures were done according to the manufacturer’s instruction. Images were taken with DMI6000B (Leica, Heidelberg, Germany) and the number of EdU- and Hoechst-positive cells number was quantified using Microsystems LAS AF-TCS MP5 (Leica). Ratio of proliferative cells was determined by the ratio of EdU-positive cells to Hoechst-positive cells. As for experiments using conditioned media, conditioned media was used to culture the cells under hypoxia for 12 h prior to EdU incorporation.

### Intracellular ROS Measurement

Cells were cultured and treated with tyrosol as described above. Intracellular ROS level was detected using the peroxide-sensitive fluorescent probe, 2′,7′-dichlorodihydrofluorescein diacetate (DCFH-DA, Beyotime, Shanghai, China) and then performed as described previously (Ariyanti et al., 2019). Briefly, cells were exposed to 20 µM (final concentration) of DCFH-DA for 30 min at 37°C. Images were taken with DMI6000B (Leica) and analyzed using ImageJ software. The results were expressed as the mean of relative fluorescence intensity per cell.

### Transwell Chamber Assay

C2C12 cells were treated by tyrosol (final concentration, 50 μg/ml) as described above. Cells were then re-seeded (7 × 10^3^ cells per chamber) in the upper chamber of a transwell plate (Corning, NY, USA), whereas normoglycemia medium was placed in the lower chamber. Cells were exposed to hypoxia for 24 h, and the migration capability was determined by staining the migrated cells in the lower chamber with crystal violet (Beyotime). Images were captured with Olympus IX71 (Olympus, Tokyo, Japan). As the control, normoglycemia medium was used, and PBS was added instead of tyrosol. For assessing the migration capability of HUVECs and MOVAS, conditioned medium was used to culture the corresponding cells and added into the lower chamber.

### Phalloidin Staining

For phalloidin staining, 1.5 × 10^4^ cells per well were seeded in a 15-mm glass bottom cell culture dish and treated with tyrosol as described above. Cell fixation was done at room temperature using 4% paraformaldehyde for 30 min. Cells were then permeabilized with 0.1% Triton X-100 diluted with PBS for 5 min, followed by blocking using 1% bovine serum albumin for 1 h. Phalloidin staining of the cells was done by incubating the samples at 37°C for 60 min with phalloidin. Images were captured with Microsystems-TCS SP5 (Leica). Results are shown as fractal dimension quantification, representing the G-actin polymerization formed from F-actin. Quantification analysis was performed using ImageJ software as described previously (Vince et al., 2008).

### Apoptosis Analysis

Cells were treated with tyrosol as described, followed by treatment with Annexin V-FITC/PI Apoptosis Detection Kit (KeyGen Biotech, Nanjing, China) according to the manufacturer’s instruction. Cells were first trypsinized, then re-suspended, and incubated in binding buffer containing Annexin V-FITC and PI at room temperature for 10 min. Flow cytometry analysis was done by using FACS Calibur (BD Biosciences, San Jose, CA). Results are shown as percentage of total apoptotic cells.

### RNA Extraction and Quantitative Reverse Transcription PCR (qRT-PCR) Analysis

Cells were treated with tyrosol as described above for 6 h, and total RNAs were extracted using Trizol (Invitrogen Life Technologies) according to the manufacturer’s instruction. Total RNA of the sample (1 μg) was reverse-transcribed into cDNA using the PrimeScript RT Reagent Kit with gDNA Eraser (Takara Bio, Dalian, China). Samples containing cDNA were subjected to qRT-PCR using SYBR Premix Ex Taq (Takara Bio) to assess the mRNA expression levels. The sequences of the HO-1 (NM_010442) primer set used for qRT-PCR are as follows: forward primer: AAGAGGCTAAGACCGCCTTC; reverse primer: CATCTGTGAGGGACTCTGGTC; the sequences for Ndufaf1 (NM_027175.4) are as follows: forward primer: TGGGGACAGTAGACAAAGTGG; reverse primer: GACAGCTTCCTCTCAAAAGCAC. β-Actin (NM_007393) was used to normalize sample amplification, and the sequences of its primer set are as follows: forward primer: AGATGTGGATCAGCAAGCAG; reverse primer: GCGCAAGTTAGGTTTTGTCA. Results are shown as relative to the mRNA expression level of the corresponding controls, which are assumed as 1. For assessing the efficacy of shRNA vectors against HO-1, cells were transfected with the indicated vectors, and RNA extraction was performed after 36 h puromycin selection.

### Western Blotting and Immunofluorescence Staining

Western blotting and immunofluorescence staining were performed as described previously (Zhang et al., 2017). The antibodies used are listed in **Supporting Table S1**. For Western blotting, β-actin was used as a loading control. Quantitative analysis of Western blotting results was done using Quantity One (Thermo Scientific, Waltham, MA); results were displayed as relative expression levels by normalizing the results to the expression levels of control, which were assumed as 1.

### Mitochondrial Membrane Potential and ROS Stainings

Cells were cultured and treated with tyrosol as described above. Mitochondrial membrane potential and ROS were stained using MitoTracker Red CMXRos and MitoSOX Red Mitochondrial Superoxide Indicator (both from Yeasen Biotech, China), respectively. Cells were labeled with MitoTracker Red CMXRos (final concentration, 100 nM) or MitoSOX Red (final concentration, 3 μM) at 37°C for 30 min. The cells were then fixed with 4% paraformaldehyde for 15 min, followed by nuclei staining using DAPI (Beyotime). Images were taken with DMI6000B (Leica) and fluorescence intensity was measured using ImageJ software. The results were expressed as the mean of relative fluorescence intensity per cell.

### Statistical Analysis

Statistical analysis was performed using Student’s *t*-test unless otherwise stated. *P < 0.05 was regarded as significant, whereas **P < 0.01 was regarded as highly significant.

## Results

### Tyrosol Increases the Survival and Proliferation of Skeletal Muscle Cells Under Hyperglycemia-Induced Oxidative Stress

**Figure 1:**
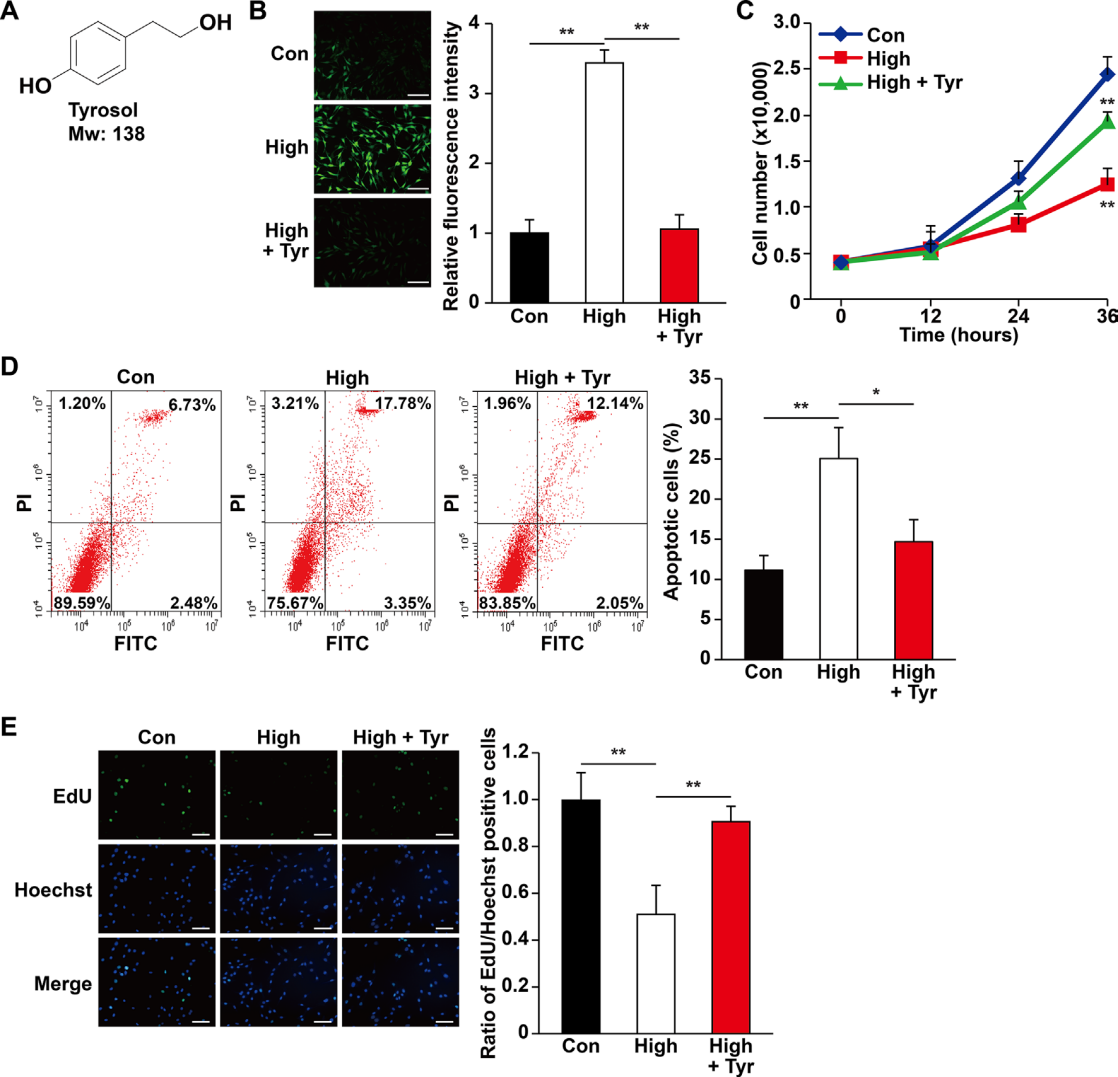
Tyrosol increases skeletal muscle cells viability by repressing hyperglycemia-induced intracellular ROS level. **(A)** Chemical structures of tyrosol. **(B)** Intracellular reactive oxygen species (ROS) levels in C2C12 cells cultured under hyperglycemia and treated with tyrosol, as detected by DCFH-DA staining; left, representative images (scale bars, 200 µm); right, relative fluorescence intensity quantification (n = 6). **(C)** Total cell number of C2C12 cells cultured under hyperglycemia and treated with tyrosol at the designated time points. **(D)** Percentage of apoptotic C2C12 cells cultured under hyperglycemia and treated with tyrosol. Cells were labeled Annexin-V-FITC/PI, and the percentage of apoptotic cells was analyzed using FACS; left, representative images; right, average percentage of apoptotic cells (n = 3). **(E)** Ratio of proliferative cells in C2C12 cells cultured under hyperglycemia and treated with tyrosol. Proliferative cells were examined using EdU-incorporation assay; left, representative images (scale bars, 200 µm); right proliferative cells quantification (n = 6). All experiments were done by subjecting the cells into hypoxia. Cells treated with PBS under normoglycemia were used as control. Quantification data were shown as relative to that of control, and expressed as mean ± SD (*P < 0.05, **P < 0.01); Con, normoglycemia; High, hyperglycemia; Tyr, tyrosol.

Hyperglycemia is known to induce systemic damages, such as increased apoptosis and decreased cell proliferation in affected cells and tissues (Tepper et al., 2002; Pottecher et al., 2018). The resulting decrease in cell survival has mainly been attributed to the intracellular increase in ROS level caused by hyperglycemia-induced oxidative stress (Giacco and Brownlee, 2010; Newsholme et al., 2016). *Rhodiola*, a plant grown in high altitude, is widely known for its usage in preventing cellular damages caused by hypoxic or oxidative stress conditions. Herein, we examined whether tyrosol (**Figure 1A**), a main active compound in *Rhodiola*, could protect cells and tissue from hyperglycemia-induced oxidative stress. To evaluate the effect of tyrosol on the survival rate of skeletal muscle cells, we first examined intracellular ROS level in the tyrosol-treated skeletal muscle cell line C2C12 cultured under hyperglycemia. Our results showed that the increased hyperglycemia-induced intracellular ROS level was significantly suppressed following tyrosol treatment (**Figure 1B**). Furthermore, we observed that tyrosol treatment led to the increase of the total cell number, which had been suppressed by hyperglycemia (**Figure 1C**). These results strongly suggested that tyrosol could increase the cell viability under hyperglycemia.

Among cellular organelles, mitochondrion is the main source of ROS upon exposure to the oxidative stress. Indeed, tyrosol suppressed the level of mitochondrial ROS induced by hyperglycemia (**Figure S1A**), most plausibly by restoring the expression level of Ndufaf1 (**Figure S1B**), a member of complex I of electron transport chain whose expression is disrupted in HO-1^-/-^ mice (Suliman et al., 2017). Concomitantly, as shown by the mitochondrial membrane potential level, hyperglycemia disrupted mitochondrial integrity, whereas tyrosol significantly reduced this effect (**Figure S1C**).

The increase of total cell number might be reflective of decreased apoptosis, increased cell proliferation, or both. Hence, we then analyzed the apoptotic rate and proliferation capability of tyrosol-treated C2C12 cells under hyperglycemia. As shown in **Figure 1D**, hyperglycemia increased the percentage of apoptotic C2C12 cells, whereas tyrosol treatment clearly suppressed both early (lower right phase) and late (upper right phase) apoptotic events. Tyrosol treatment also grossly enhanced cell proliferation, indicated by increased ratio of EdU-positive cells (**Figure 1E**). Overall, these results indicated that tyrosol increased the survival rates and proliferation of skeletal muscle cells, most plausibly by reducing hyperglycemia-induced intracellular ROS level.

### Tyrosol Promotes Cell Viability by Inducing HO-1 Expression

**Figure 2:**
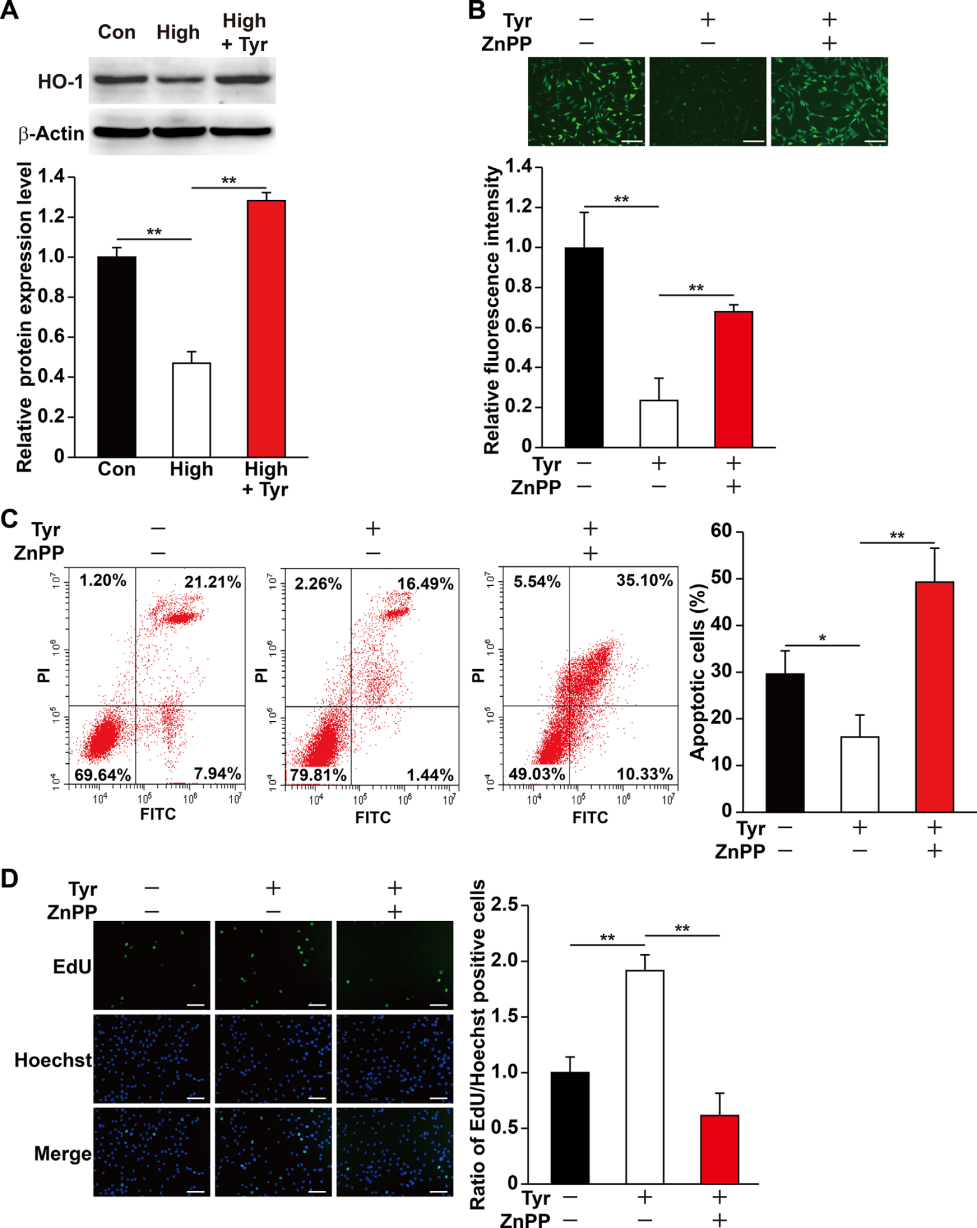
Tyrosol increases skeletal muscle cells viability under hyperglycemia by inducing HO-1 expression. **(A)** Protein expression level in C2C12 cells cultured under hyperglycemia and treated with tyrosol, as analyzed by Western blotting using corresponding antibodies; upper panels, representative images; lower panels, quantification results (n = 3). β-Actin was used as loading control. Cells treated with PBS under normoglycemia were used as control. **(B)** Intracellular ROS levels in C2C12 cells treated with tyrosol and zinc protoporphyrin IX (ZnPP; final concentration, 10 µM), as detected by DCFH-DA staining; upper panels, representative images (scale bars, 200 µm); lower panels, relative fluorescence intensity quantification (n = 6). Cells treated with DMSO under hyperglycemia were used as control. **(C)** Percentage of apoptotic cells in C2C12 cells cultured under hyperglycemia and treated with tyrosol and ZnPP (final concentration, 10 µM). Cells were labeled with Annexin-V-FITC/PI, and the percentage of apoptotic cells were analyzed using FACS; left, representative images; right, average percentage of apoptotic cells (n = 3). Cells treated with DMSO under hyperglycemia were used as control. **(D)** Ratio of proliferative cells in C2C12 cells cultured under hyperglycemia and treated with tyrosol and ZnPP (final concentration, 10 µM). Proliferative cells were examined using EdU-incorporation assay; left, representative images (scale bars, 200 µm); right, the quantitative results (n = 6). Cells treated with DMSO under hyperglycemia were used as control. All experiments were done by subjecting the cells into hypoxia. Quantification data were shown as relative to that of control, and expressed as mean ± SD (*P < 0.05, **P < 0.01); Con, normoglycemia; High, hyperglycemia; Tyr, tyrosol.

HO-1 is known as a cytoprotective factor. Knocking down HO-1 (**Figures S2**, **S3**) significantly promoted intracellular ROS level in C2C12 cells cultured under hyperglycemia (**Figure S4**). Given that tyrosol could suppress the damage caused by hyperglycemia-induced intracellular ROS, we examined the role of HO-1 in tyrosol-induced cytoprotection. Quantitative RT-PCR and Western blot analysis showed that although hyperglycemia suppressed the expression of HO-1 in C2C12 cells, tyrosol treatment conspicuously enhanced it (**Figure 2A** and **S5**). We then used HO-1 inhibitor zinc protoporphyrin IX (ZnPP) to block the HO-1 pathway and observed that ZnPP significantly restored intracellular ROS level that had been suppressed by tyrosol treatment (**Figure 2B**). ZnPP also restored the hyperglycemic-induced increase in apoptotic rate and decrease in proliferation capability, nullifying the effects of tyrosol treatment on C2C12 cells cultured under hyperglycemia (**Figures 2C, D**). Overall, these results proved the cytoprotective effect of tyrosol on skeletal muscle cells under hyperglycemia, which can be attributed to the enhanced expression of HO-1, which in turn suppressed intracellular ROS level.

### Tyrosol Improves the Secretory and Migration Capabilities of Skeletal Muscle Cells Under Hyperglycemia

**Figure 3:**
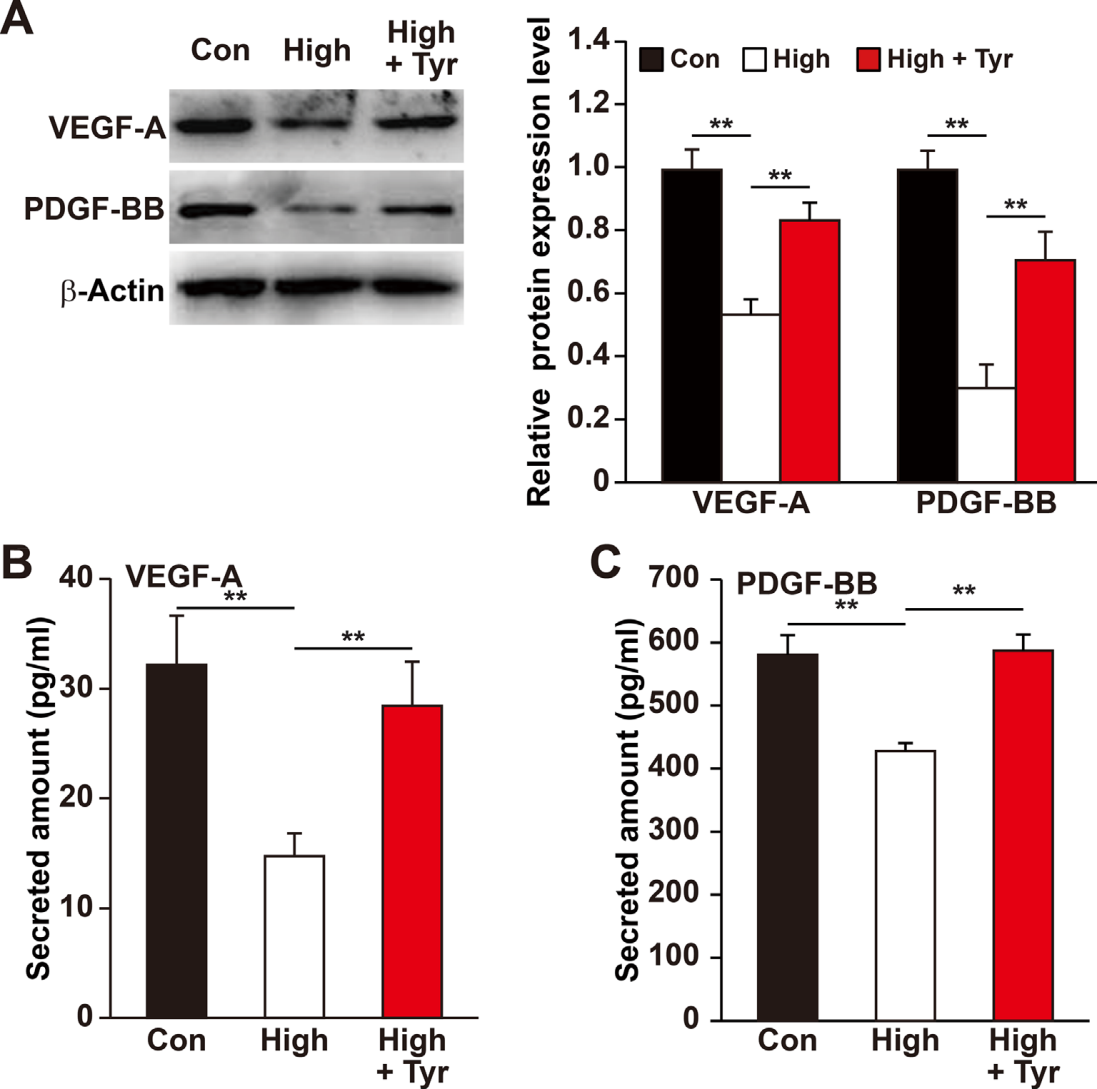
Tyrosol enhances VEFG-A and PDGF-BB secretion from skeletal muscle cells under hyperglycemia. **(A)** Protein expression levels of VEGF-A and PDGF-BB in C2C12 cells cultured under hyperglycemia and treated with tyrosol; left, representative images; right, quantification results. **(B)** Secretion level of VEGF-A in C2C12 cells cultured under hyperglycemia and treated with tyrosol, as determined by ELISA. **(C)** Secretion level of PDGF-BB in C2C12 cells cultured under hyperglycemia and treated with tyrosol, as determined by ELISA. All experiments were done by subjecting the cells into hypoxia; cells treated with PBS under normoglycemia were used as control. Quantification data were shown as relative to that of control, and expressed as mean ± SD (n = 3). **P < 0.01; Con, normoglycemia; High, hyperglycemia; Tyr, tyrosol.

Previous reports revealed that the ability of skeletal muscle cells to express and secrete various angiogenic factors make them crucial for angiogenesis (Tateno et al., 2006; Wu et al., 2015) and that hyperglycemia disrupts this paracrine function (D’Souza et al., 2013; Ciaraldi et al., 2016; Monaco et al., 2017). Of all the angiogenic factors, VEGF-A plays a crucial role in tube formation, whereas PDGF-BB takes part in the maturation of newly formed blood vessels (Lindahl et al., 1997; Tanii et al., 2006; Carmeliet and Jain, 2011). Therefore, we analyzed if tyrosol is capable in promoting this function of the skeletal muscle cells under hyperglycemic stress. As shown in **Figure 3A**, tyrosol treatment could induce VEGF-A and PDGF-BB expressions in C2C12 cells, which were suppressed under hyperglycemia, to a level similar to those observed in the normoglycemic control. In line with this, the secreted level of VEGF-A and PDGF-BB from tyrosol-treated C2C12 cells also demonstrated a significant increase (**Figures 3B, C**).

**Figure 4:**
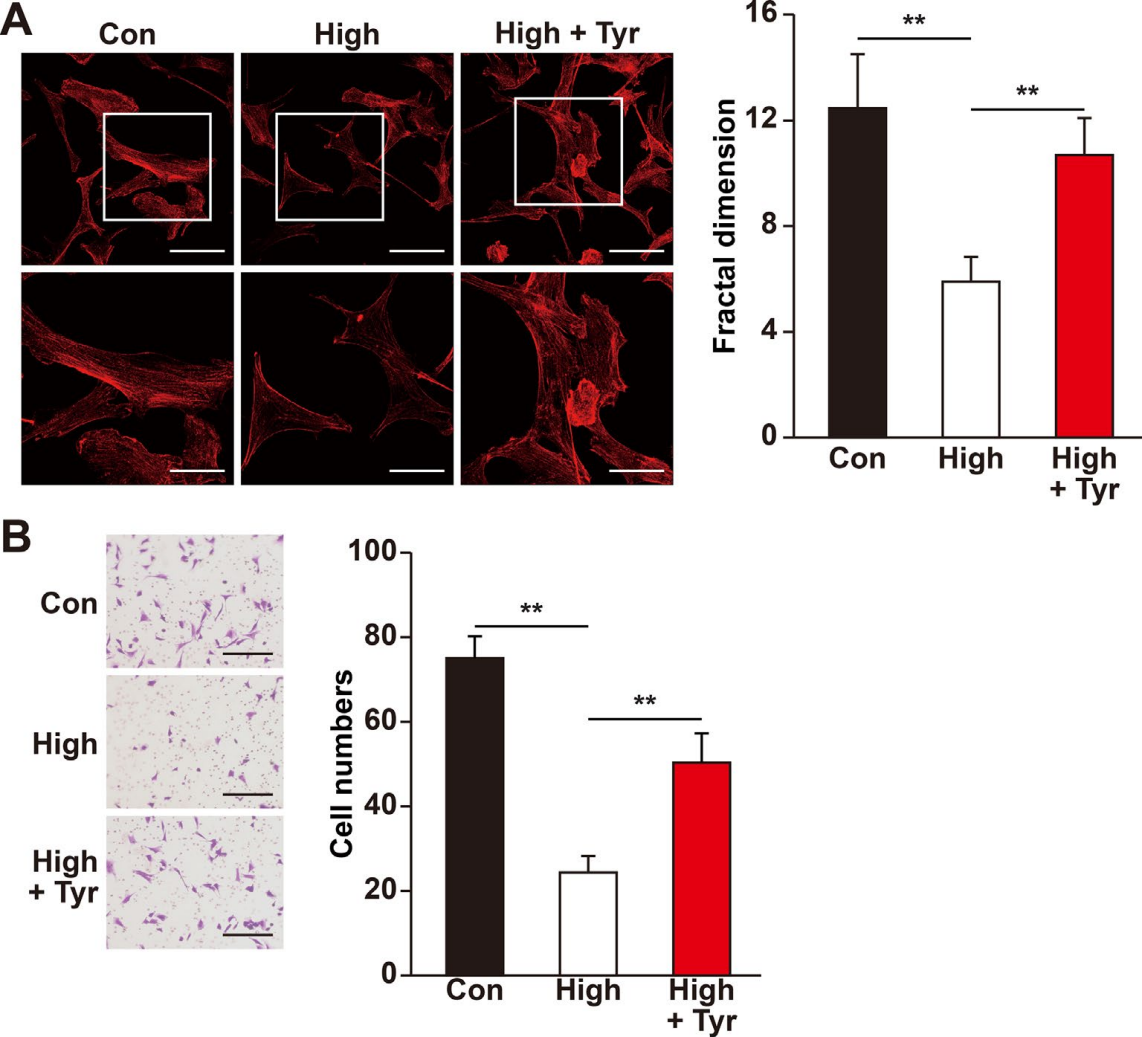
Tyrosol restores migration capability of skeletal muscle cells suppressed by hyperglycemia. **(A)** Morphological changes of F-actin in C2C12 cells treated with tyrosol and cultured under hyperglycemia; left, representative images (scale bars, 100 µm for upper panels and 50 µm for lower panels; lower panels show the high magnification images of the cropped part in the upper panels); right, quantification analysis of fractal dimension. **(B)** Migration capability in C2C12 cells cultured under hyperglycemia and treated with tyrosol, as analyzed using transwell chamber assay; left, representative images (scale bars, 100 µm); right, quantification of migrated cells. All experiments were done by subjecting the cells into hypoxia. Quantification data were expressed as mean ± SD (n = 6; **P < 0.01); Con, normoglycemia; High, hyperglycemia; Tyr, tyrosol.

Recent studies showed that skeletal muscle cell migration capability is another critical factor that promotes angiogenesis, most plausibly by disseminating the secreted angiogenic factors into a larger area (Tateno et al., 2006; Zhang et al., 2017). Hence, we also analyzed if tyrosol could increase skeletal muscle cell migration capability. The results of phalloidin staining showed that tyrosol treatment significantly induced the polymerization of G-actin into F-actin, as well as the formation of pseudopodia, which are necessary for cell migration (**Figure 4A**). Concomitantly, the transwell chamber assay demonstrated that tyrosol treatment increased the number of C2C12 cells in the lower chamber, indicating the migration capability of the cells in response to tyrosol treatment (**Figure 4B**). Together, these results elucidated novel functions of tyrosol in enhancing the secretion and migration of skeletal muscle cells, which were impaired under hyperglycemia.

### Tyrosol Activates the Proliferation Capability of Vascular Endothelial and Smooth Muscle Cells Through the Secretome of Skeletal Muscle Cells

**Figure 5:**
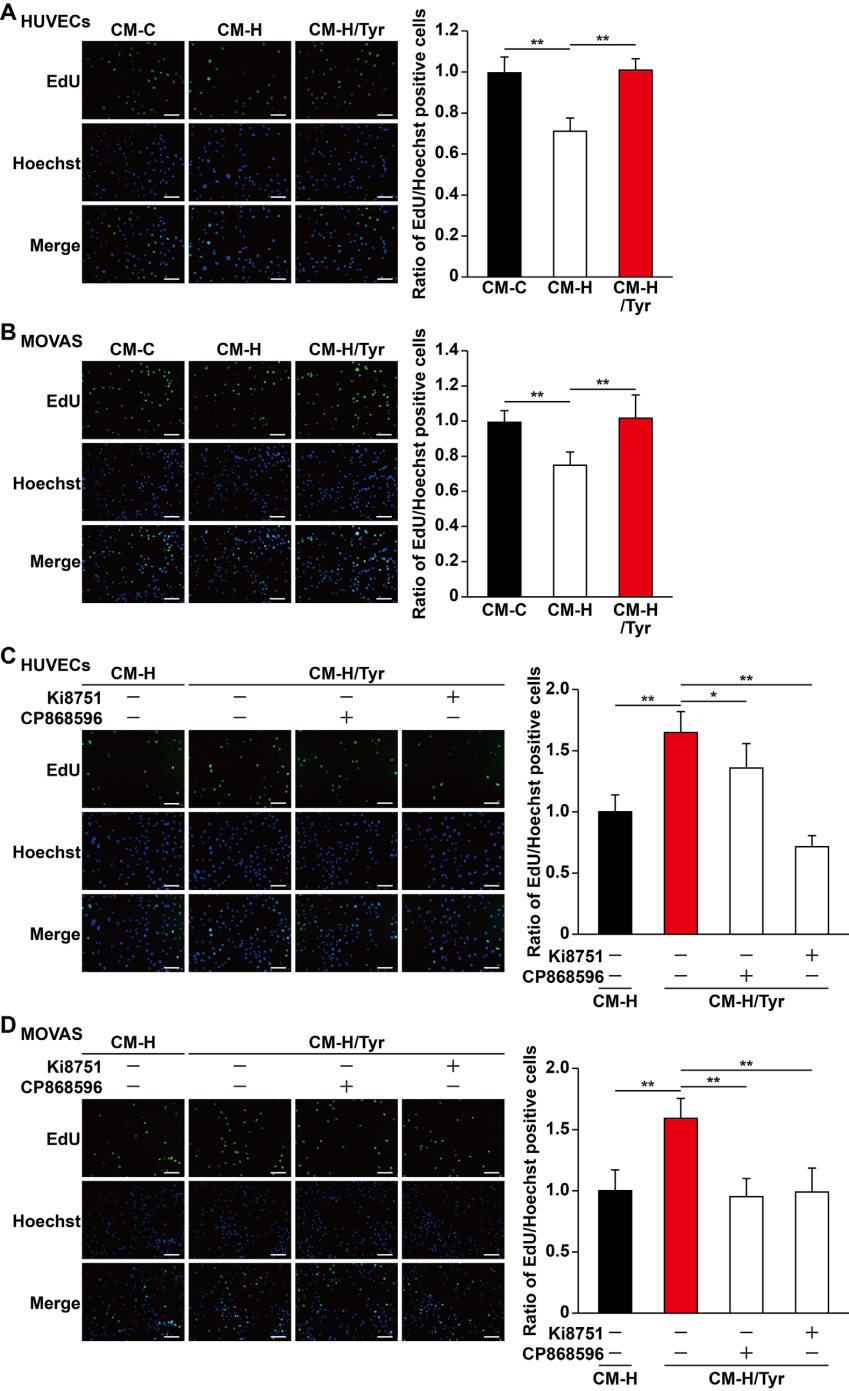
Tyrosol-treated skeletal muscle cells’ secretome promotes vascular endothelial and smooth muscle cells proliferation capability suppressed by hyperglycemia. **(A–B)** Ratio of proliferative cells in HUVECs **(A)** and MOVAS cells **(B)** cultured with conditioned media collected from tyrosol-treated C2C12 cells. Proliferative cells were examined using EdU-incorporation assay; left, representative images; right, the quantitative results. **(C–D)** Ratio of proliferative cells in HUVECs **(C)** and MOVAS cells **(D)** cultured with conditioned media collected from tyrosol-treated C2C12 cells and treated with VEGFR inhibitor (Ki8751; final concentration, 2 nM) or PDGFR-β inhibitor (CP868596; final concentration, 0.3 µM). Proliferative cells were examined using EdU-incorporation assay; left, representative images; right, the quantitative results. Scale bars, 200 µm. All experiments were done by subjecting the cells into hypoxia. Quantification data were expressed as mean ± SD (n = 6; *P< 0.05, **P < 0.01); CM-C, conditioned medium derived from C2C12 cells treated with PBS under normoglycemia; CM-H, conditioned medium derived from C2C12 cells treated with PBS under hyperglycemia; CM-H/Tyr, conditioned medium derived from C2C12 cells treated with tyrosol under hyperglycemia.

Mature blood vessels formation is crucial to yield an efficient enhancement of blood perfusion. Herein, vascular endothelial cells are ensheathed by smooth muscle cells to make up mature blood vessels (Jain, 2003; Semenza, 2003). Although induction of single angiogenic factor often results in leaky vessels formation, paracrine signals from skeletal muscle cells, especially of those major angiogenic factors (e.g., VEGF-A and PDGF-BB), have been shown to positively influence the potential of vascular endothelial and smooth muscle cells, subsequently promoting the formation of mature blood vessels (Perez-Ilzarbe et al., 2008; Jazwa et al., 2016; Gianni-Barrera et al., 2018). As shown in above results, tyrosol improved the viability and migration capabilities of skeletal muscle cells, as well as their function in expressing and secreting VEGF-A and PDGF-BB, which were disrupted in hyperglycemia. These results indicated that tyrosol might affect vascular endothelial and smooth muscle cells under hyperglycemia through paracrine mechanism exerted by skeletal muscle cells. To elucidate this potential of tyrosol, we prepared the conditioned media from tyrosol-treated C2C12 cells. Treatment was done by culturing C2C12 cells with tyrosol under hyperglycemia (CM-H/Tyr). As a comparison, C2C12 cells were treated with PBS and cultured under normoglycemic (CM-C) or hyperglycemic conditions (CM-H). To obtain the conditioned media, C2C12 cells were washed after treatment with tyrosol and incubated with a new medium to eliminate the possibility of tyrosol carryover. When cultured with CM-H, we found that HUVECs exhibited a significantly decreased EdU-positive cell ratio than those cultured with CM-C, whereas treatment of HUVECs with CM-H/Tyr could restore the ratio of EdU-positive cells (**Figure 5A**). Similar results were obtained in MOVAS cells cultured with CM-H/Tyr (**Figure 5B**). These results indicated that secreted factors of C2C12 cells treated with tyrosol could replenish the proliferation rate of the vascular endothelial and smooth muscle cells, which were suppressed by hyperglycemia-induced abnormal secretome levels in skeletal muscle cells.

Next, we also attempted to further elucidate the mechanism on how tyrosol-induced secreted factors from skeletal muscle cells could enhance the proliferation of vascular endothelial and smooth muscle cells. Given that the aforementioned factors, VEGF-A and PDGF-BB, are critical for cell proliferation, we then blocked their receptor-binding pathways using VEGF-A receptor inhibitor Ki8751 and PDGF-BB receptor inhibitor CP868596. Both Ki8751 and CP868596 significantly suppressed the proliferation capability of HUVECs and MOVAS cells, which was initially restored in CM-H/Tyr (**Figure 5C, D**). Overall, these results suggested that VEGF-A/VEGFR and PDGF-BB/PDGFR-β pathways play a significant role in mediating the enhancement of vascular endothelial and smooth muscle cells proliferation upon treatment with the secretome of tyrosol-treated skeletal muscle cells.

### Tyrosol Enhances the Migration Capabilities of the Vascular Endothelial and Smooth Muscle Cells Through the Secretome of Skeletal Muscle Cells

**Figure 6:**
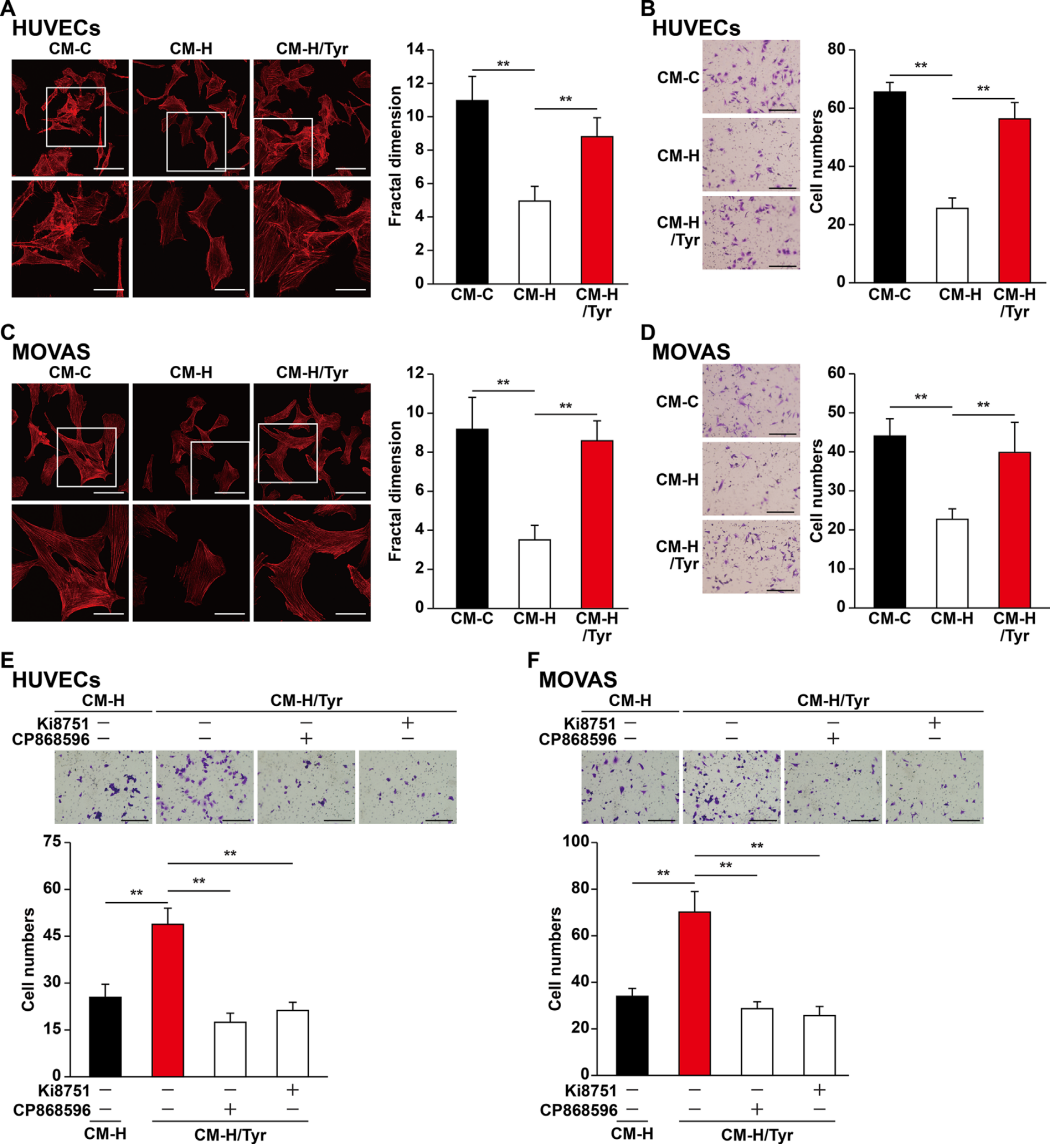
Tyrosol-treated skeletal muscle cells’ secretome promotes vascular endothelial and smooth muscle cells migration capability suppressed by hyperglycemia. **(A)** Morphological changes of F-actin in HUVECs cultured with conditioned medium collected from tyrosol-treated C2C12 cells; left, representative images (scale bars, 100 µm for upper panels and 50 µm for lower panels; lower panels show the high-magnification images of the cropped part in the upper panels); right, quantification analysis of fractal dimension. **(B)** Migration capability in HUVECs cultured with conditioned medium collected from tyrosol-treated C2C12 cells, as analyzed using transwell chamber assay; left, representative images (scale bars, 100 µm); right, the quantitative results. **(C)** Morphological changes of F-actin in MOVAS cells cultured with conditioned medium collected from tyrosol-treated C2C12 cells; left, representative images (scale bars, 100 µm for upper panels and 50 µm for lower panels; lower panels show the high magnification images of the cropped part in the upper panels); right, quantification analysis of fractal dimension. **(D)** Migration capability in MOVAS cells cultured with conditioned medium collected from tyrosol-treated C2C12 cells, as analyzed using transwell chamber assay; left, representative images (scale bars, 100 µm); right, the quantitative results. **(E**–**F)** Migration capability in HUVECs **(E)** and MOVAS cells **(F)** cultured with conditioned medium collected from tyrosol-treated C2C12 cells and Ki8751 (final concentration, 2 nM) or CP868596 (final concentration, 0.3 µM), as analyzed using transwell chamber assay; upper panels, representative images (scale bars, 100 µm); lower panels, quantification results. All experiments were done by subjecting the cells into hypoxia. Quantification data were expressed as mean ± SD (*n* = 6; **P < 0.01); CM-C, conditioned medium derived from C2C12 cells treated with PBS under normoglycemia; CM-H, conditioned medium derived from C2C12 cells treated with PBS under hyperglycemia; CM-H/Tyr, conditioned medium derived from C2C12 cells treated with tyrosol under hyperglycemia.

Besides their proliferation capability, previous studies also indicate that the migration capability of vascular endothelial and smooth muscle cells toward ischemic sites is critical for angiogenic efficacy (Gerthoffer, 2007; Zhang et al., 2017). We found that CM-H/Tyr grossly promoted F-actin polymerization and the number of pseudopodia in HUVECs (**Figure 6A**), as well as the number of HUVECs migrated to the lower chamber (**Figure 6B**). Similarly, CM-H/Tyr enhanced F-actin polymerization in MOVAS cells, as well as their migration capability (**Figures 6C, D**).

To elucidate the molecular mechanism by which CM-H/Tyr induced the migration capability of vascular endothelial and smooth muscle cells, we blocked the VEGF-A and PDGF-BB pathways using Ki8157 and CP868596, respectively. Disrupting these pathways diminished the CM-H/Tyr-restored migration capability (**Figures 6E, F**). Collectively, these results demonstrated that VEGF-A/VEGFR and PDGF-BB/PDGFR-β pathways are critical for upregulating the migration capability of vascular endothelial and smooth muscle cells upon treatment with the secretome of tyrosol-treated skeletal muscle cells.

### Tyrosol Enhances Blood Perfusion Recovery in the Diabetic HLI Model Mice

**Figure 7:**
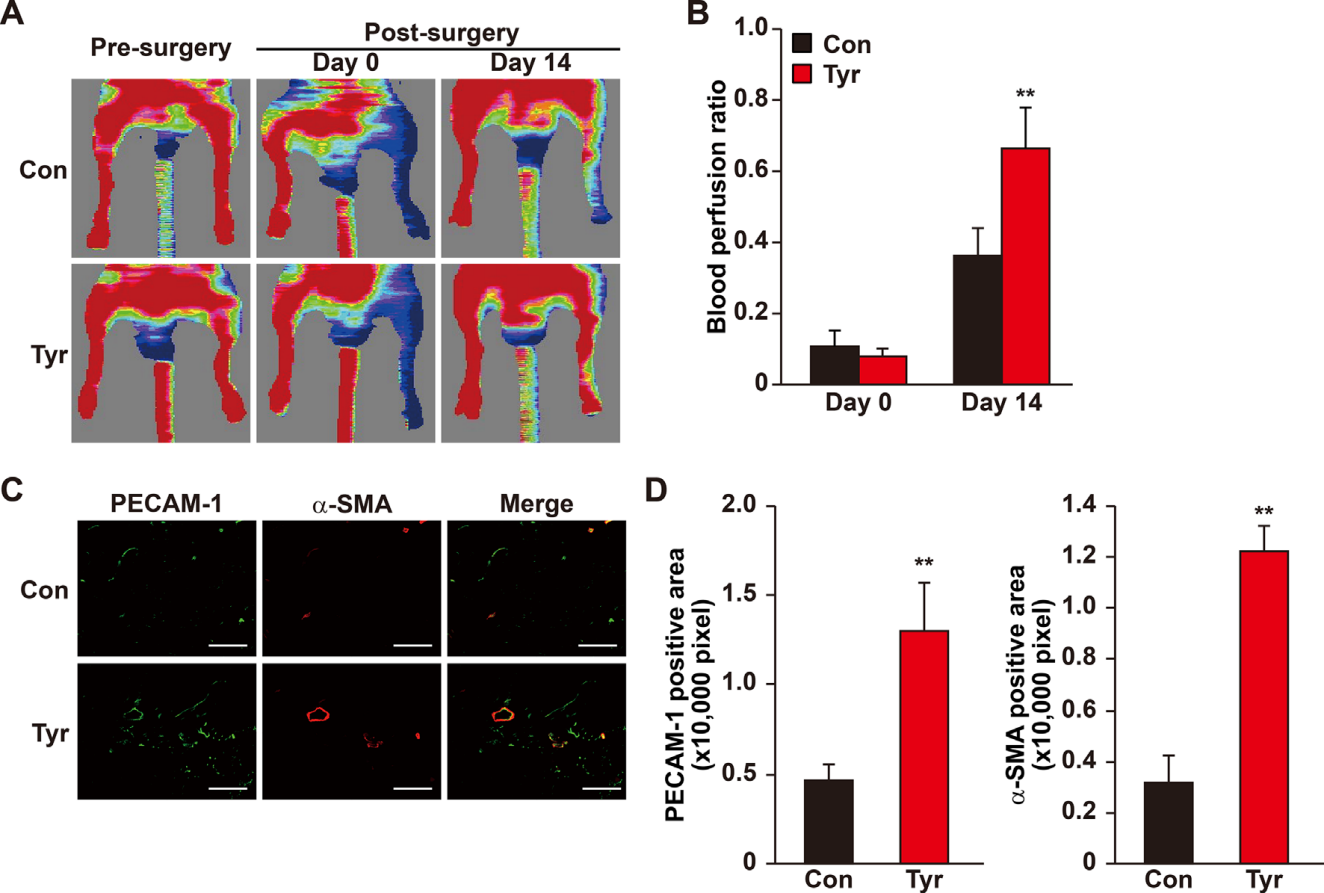
Tyrosol induces mature blood vessels formation and blood perfusion recovery in diabetic HLI model mice. **(A**–**B)** Blood perfusion recovery in the ischemic and non-ischemic hindlimb of diabetic HLI model mice injected by tyrosol: representative of Laser Doppler Perfusion Images **(A)**; and quantification of the blood perfusion ratio at day 0 and day 14 **(B)** (n = 5, *P* value was analyzed by one-way ANOVA) were shown. **(C**–**D)** Immunofluorescence staining of PECAM-1 (green) and α-SMA (red) in the gastrocnemius muscle of the ischemic hindlimbs of diabetic HLI model mice injected by tyrosol at 14 days post-surgery. Representative images (**C**; scale bars, 200 µm); and quantification results of PECAM-1 and α-SMA-positive areas (**D**; n = 6) were shown. Tyrosol and PBS were administered by local injection intramuscularly. Quantification data were expressed as mean ± SD (**P < 0.01); Con, mice injected with PBS; Tyr, mice injected with tyrosol.

Our results showed that angiogenic factors secreted by tyrosol-treated skeletal muscle cells significantly enhanced the proliferation and migration capabilities of vascular endothelial and smooth muscle cells. Given that these regulations are crucial for angiogenesis, these results suggested the possibility of using tyrosol to enhance new blood vessels formation in diabetic HLI patients. However, the bioavailability of orally administered tyrosol is very low due to the first-pass effect, and furthermore, tyrosol is eliminated rapidly from most organs and tissues (Lee et al., 2016). Thus, we established a mouse model for diabetic HLI and treated the mice with intramuscular administration of tyrosol. Furthermore, to ensure that sufficient amount of tyrosol could be distributed to the skeletal muscle cells, intramuscular injection was performed every 3 days. As depicted in **Figures 7A, B**, enhanced blood perfusion was shown in the ischemic hindlimb of tyrosol-treated diabetic mice, reaching almost 70% at 14 days after treatment, whereas for the PBS-treated control group, the recovery rate was about 35%. Furthermore, immunofluorescence staining against platelet endothelial cell adhesion molecule-1 (PECAM-1) and alpha-smooth muscle actin (α-SMA), representing vascular endothelial and smooth muscle cells respectively, revealed a notable increase in the PECAM-1-positive cells surrounded by α-SMA-positive cells, which demonstrates mature blood vessels (**Figures 7C, D**). These results clearly indicated that tyrosol treatment promoted angiogenesis in diabetic HLI mice.

**Figure 8:**
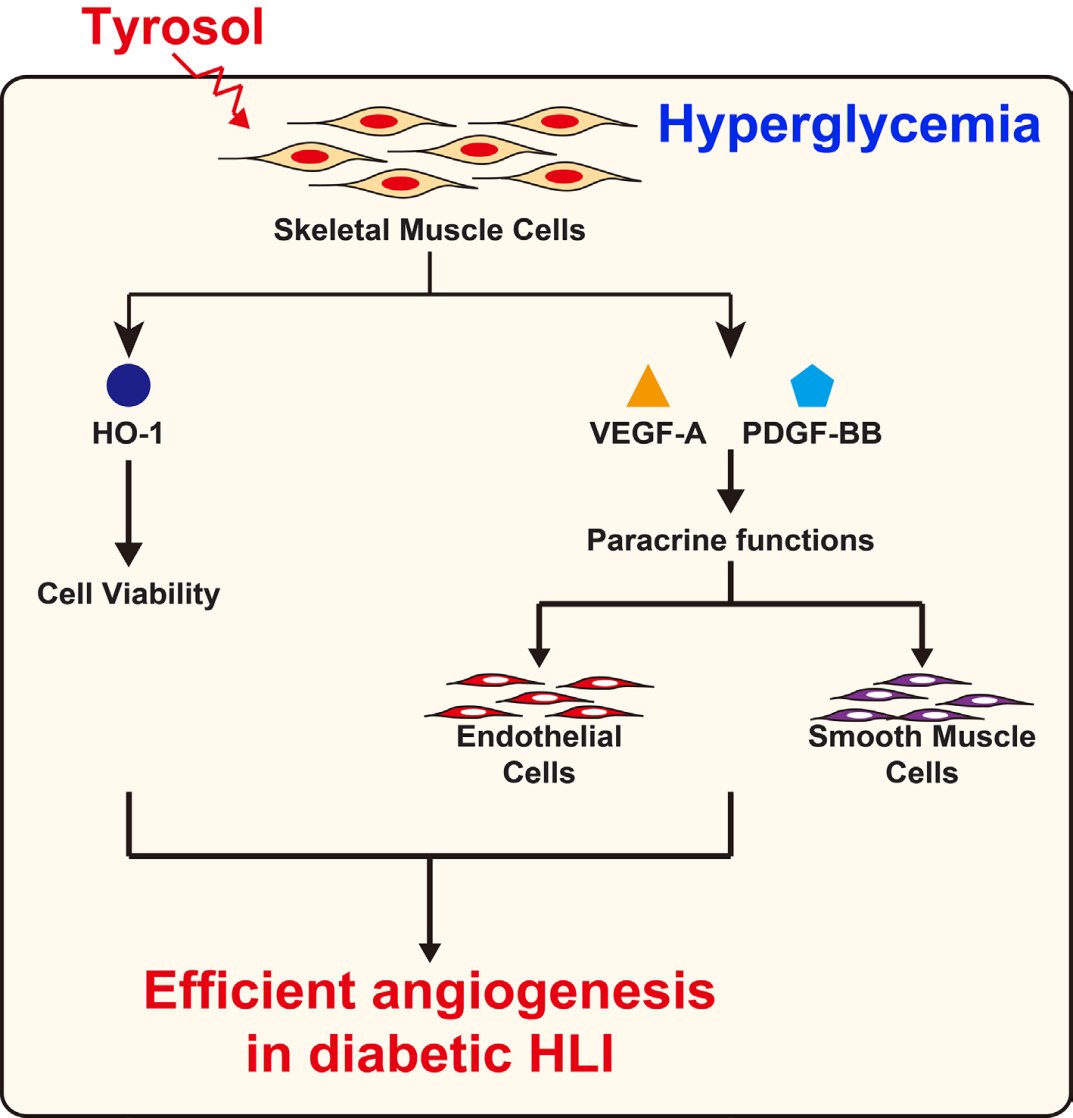
Schematic diagram of the effect of tyrosol in promoting angiogenic capability in diabetic HLI.

Overall, our results demonstrated that intramuscular tyrosol administration could induce the formation of mature, functional blood vessels in diabetic HLI mice, plausibly by enhancing the expression of HO-1 thereby protecting skeletal muscle cells from hyperglycemia-induced oxidative stress, leading to an increase in skeletal muscle cell number, secreted angiogenic factors, and migration capability. These tyrosol-induced effects in turn positively regulate the proliferation and migration capabilities of vascular endothelial and smooth muscle cells, especially through the VEGF-A/VEGFR and PDGF-BB/PDGFR-β axis (**Figure 8**).

## Discussion

The ability of the skeletal muscle to secrete various angiogenic factors simultaneously makes it a prospective target for inducing effective therapeutic angiogenesis, a potential strategy for treating arterial diseases, such as HLI, which aims to enhance the formation of functional blood vessels. The previous studies showed that targeting skeletal muscle cells could significantly increase blood perfusion recovery in the HLI mice (Tateno et al., 2006; Wu et al., 2015; Zhang et al., 2017). However, hyperglycemia-induced systemic impairment led to the disruption of the paracrine and other cellular functions of skeletal muscle cells (Ciaraldi et al., 2016; Monaco et al., 2017). This results in the aberrant decrease on angiogenic factors expression, subsequently deteriorating the endogenous angiogenesis potential in diabetic HLI patients (Perez-Ilzarbe et al., 2008; D’Souza et al., 2013; Lizotte et al., 2013). Thus, the defect in endogenous angiogenesis potential is the obstacle for applying therapeutic angiogenesis strategy in diabetic HLI (Thangarajah et al., 2009; Jude et al., 2010; Rask-Madsen and King, 2013), and stimulating this potential is the key for successful therapeutic angiogenesis for diabetic HLI patients.

Herein, we showed that hyperglycemia suppresses skeletal muscle cells viability by promoting intracellular ROS level, thereby inducing their apoptosis, and simultaneously suppressing their proliferation capability; while tyrosol restores these capabilities. Furthermore, this effect of tyrosol was plausibly due to its capability to induce the expression of HO-1 under hyperglycemia. HO-1 is a cell survival factor that suppresses oxidative stress-induced cellular damage and mitigates cellular injury by exerting anti-oxidant, anti-apoptotic, and anti-inflammatory effects, and insufficient HO-1 levels lead to an enhanced apoptosis rate (Di Filippo et al., 2005; Araujo et al., 2012; Gou et al., 2018). Mitochondrion is the main source of ROS upon exposure to the oxidative stress, such as those caused by hyperglycemia. Indeed, hyperglycemia disrupts skeletal muscle cells’ mitochondrial function and increases mitochondrial ROS level (Pottecher et al., 2018). Tyrosol restored the expression of Ndufaf1, a member of complex I of electron transport chain under hyperglycemia. This leads to the suppression of mitochondrial ROS level under hyperglycemia, and subsequently, the reduction of hyperglycemia-induced mitochondrial damage.

We also found that tyrosol restores the paracrine function of skeletal muscle cells suppressed by hyperglycemia. This enables tyrosol to increase VEGF-A and PDGF-BB secretion, which are crucial for functional angiogenesis. Moreover, tyrosol also recovers the migration capability of skeletal muscle cells under hyperglycemia, most plausibly by enhancing G-actin polymerization to form F-actin. In response to angiogenic stimuli exerted by skeletal muscle cells, responsible cells in forming mature blood vessels, i.e., vascular endothelial and smooth muscle cells, proliferate and expand their pseudopodia to migrate toward the stimuli (Gerthoffer, 2007; Lamalice et al., 2007). Furthermore, as the migration capability of skeletal muscle cells are significantly improved, the dispersion area of secreted angiogenic stimuli becomes wider, leading to a more efficient angiogenesis induction (Tateno et al., 2006; Zhang et al., 2017).

Angiogenesis is an intricate process involving various factors and types of cells, making it harder to be recapitulated. Attempts using single angiogenic factor for therapeutic angiogenesis have been made; however, these approaches could not mimic an efficient neovascularization (Hendel et al., 2000; Celletti et al., 2001; Epstein et al., 2001; Khurana et al., 2005). Hence, currently, the application of combined several angiogenic factors has been considered more favorable (Cao et al., 2003; Kano et al., 2005). Thus, tyrosol-induced recovery of secreted VEGF-A and PDGF-BB levels in skeletal muscle cells, which was suppressed by hyperglycemia, is particularly important for therapeutic angiogenesis. These secreted factors mediate the communication between skeletal muscle cells and blood vessels forming cells through the activated VEGF-A/VEGFR and PDGF-BB/PDGFR-β pathway. VEGF-A is important for tube formation, as it enhances the proliferation and migration capability of vascular endothelial cells, and its impaired expression is closely related with the dysfunction of endothelial cells (Avogaro et al., 2011; Caporali et al., 2015). PDGF-BB, whose expression is disrupted during hyperglycemia by the increase of advanced glycation end products (AGE), is crucial for the maturation of blood vessels formed (Tanii et al., 2006), as it enhances the migration capability of endothelial cells, smooth muscle cells, and pericytes (Xue et al., 2011; Ge et al., 2015). Although further investigation is required to ascertain if tyrosol also affects the levels of other angiogenic factors, its therapeutic effect on diabetic HLI model mice could be attributed, at least partly, to the increased secretion of VEGF-A and PDGF-BB.

Tyrosol is a major compound of *Rhodiola*, which has been used as Chinese traditional medicine for centuries (Gupta et al., 2008; Chiang et al., 2015; Torrens-Spence et al., 2018). *Rhodiola* is known to enhance adaptation to high-altitude and hypoxic conditions, as it promotes erythropoietin (EPO) expression and cell survival (Zhang et al., 2009; Zheng et al., 2012). Apart from being a component of *Rhodiola* crude extract, tyrosol is also a metabolite of salidroside, another major compound of *Rhodiola* (Guo et al., 2014). In previous studies, we showed that salidroside could increase hypoxia-inducible factor-1α (HIF-1α) protein and subsequently induce angiogenesis (Zhang et al., 2017). In the present study, we show that tyrosol could also promote angiogenesis in diabetic HLI model mice. These results, therefore, indicate that these major components of *Rhodiola* extract may equally possess therapeutic angiogenic functions against diabetic HLI, which also indicates the holistic function of phytomedicine.

Previous studies report that oral administration of tyrosol decreases plasma glucose levels while increasing plasma insulin levels (Chandramohan et al., 2015; Chandramohan et al., 2017). However, orally administered tyrosol has very low bioavailability due to the first-pass effect. Furthermore, tyrosol is absorbed and metabolized within 1 h after administration. The metabolites are then distributed in most organs and tissues, eliminated within 4 h, and subsequently, excreted in urine (Lee et al., 2016). Therefore, in this study, we administered tyrosol once every 3 days directly into the skeletal muscle cells close to the ischemic site. Our method enables tyrosol to directly target the skeletal muscle cells, hence increasing the local concentration of angiogenic factors in ischemic tissue. Indeed, we found that blood glucose levels did not significantly change before and after tyrosol intramuscular administration (**Supporting Table S2**). These facts indicate that tyrosol-induced therapeutic angiogenesis demonstrated in this study is due to its direct effects on skeletal muscle cells, that is, protecting skeletal muscle cells from oxidative damage and enhancing the paracrine function of skeletal muscle cells, rather than on decreasing the blood glucose concentration as achieved by oral administration.

Intriguingly, an analog of tyrosol, hydroxytyrosol [2-(3,4-dihydroxyphenyl)ethanol], exerts an opposite function. Fortes et al. showed that only treatment with hydroxytyrosol, but not tyrosol, suppressed endothelial cells proliferation, migration, and tube-like formation, and subsequently exerts anti-angiogenic effect. They suggested that this difference is due to the hydroxyl group at the position 3 of the phenyl ring of hydroxytyrosol (Fortes et al., 2012). A previous study has shown that phenolic compounds, including tyrosol and hydroxytyrosol, could function as an anti-oxidant and possesses ROS clearance potential. This might be due to the presence of phenolic group, which could exert electron transfer, as well as metal chelation (Briante et al., 2003; Leopoldini et al., 2004). However, the anti-oxidant property of tyrosol, which only has one phenolic group, is far less effective, and in most cases could not exhibit any activity (Rietjens et al., 2007). In this study, we showed that tyrosol suppresses skeletal muscle cells oxidative damage and thereby increasing angiogenesis not through a direct clearance of ROS, but through enhancing the expression level of cytoprotective factor HO-1. Although the molecular mechanism of HO-1 induction by tyrosol, as well as the pharmacophore of tyrosol in exerting this function, needs to be further investigated, our results described a new function of tyrosol in protecting skeletal muscle cells and inducing angiogenesis under hyperglycemia. In summary, our findings have unraveled a novel function of tyrosol in promoting angiogenesis under hyperglycemia by promoting the survival rate and paracrine function of skeletal muscle cells, at least partially by inducing the secretion of angiogenic factors VEGF-A and PDGF-BB. The present study also reveals that intramuscular tyrosol administration significantly promotes the formation of functional blood vessels, subsequently improving the recovery of blood perfusion in diabetic HLI mice. Thus, this study provides evidence that tyrosol is a prospective small molecule drug for effective therapeutic angiogenesis to combat diabetic HLI.

## Conclusion

Tyrosol intramuscular administration significantly enhances the formation of blood vessels, subsequently improving the recovery of blood perfusion in diabetic HLI mice. Mechanistically, tyrosol exerts cytoprotective function against hyperglycemia-induced oxidative stress in skeletal muscle cells, increases their proliferation vigorously, and suppresses apoptosis of skeletal muscle cells under hyperglycemia. Tyrosol also promotes skeletal muscle cells migration capability and paracrine function under hyperglycemia. These subsequently promote angiogenesis. Despite the intramuscular tyrosol injection being a promising strategy for diabetic HLI, further pre-clinical and clinical studies are required prior to the clinical use. Furthermore, we are trying to improve the structural stability of tyrosol and/or the drug delivery system to enable the reduction of repetitive administration in the future. Nevertheless, our present study highlights the potential of the pharmacological application of tyrosol as a small molecule drug for therapeutic angiogenesis in diabetic HLI patients.

## Data Availability

The datasets generated for this study are available on request to the corresponding author.

## Ethics Statement

All animal experiments were performed in the Third Military Medical University, China. The animal studies were conducted in accordance with the Guide for the Care and Use of Laboratory Animals of the Ministry of Health, China. The protocol was approved by the Laboratory Animal Welfare and Ethics Committee of the Third Military Medical University.

## Author Contributions

VK and SW conceived the project, designed the experiments, analyzed the results, and wrote the paper. JZ performed most of the experiments. DAN and ADA performed the animal experiments. OM carried out quantitative RT-PCR analysis and mitochondria analysis. GW provided technical analysis and material support. CL performed part of the cellular experiments.

## Funding

This work was supported by grants from the National Natural Science Foundation of China (81872273 and 31871367); the Natural Science Foundation of Chongqing (cstc2018jcyjAX0374 and cstc2018jcyjAX0411); and the Fundamental Research Funds for the Central Universities (2019CDQYSW010). We also thank for the support from the State and Local Joint Engineering Laboratory for Vascular Implants (Chongqing).

## Conflict of Interest Statement

A patent related to the results of this study has been filed with Chinese patent application No. 201810173573.2.

The authors declare that the research was conducted in the absence of any commercial or financial relationships that could be construed as a potential conflict of interest.
