# Supplementary material for: Tyrosol Facilitates Neovascularization by Enhancing Skeletal Muscle Cells Viability and Paracrine Function in Diabetic Hindlimb Ischemia Mice
Source: Front Pharmacol. 2019 Aug 14;10:909. doi: 10.3389/fphar.2019.00909 (PMC6702659; doi:10.3389/fphar.2019.00909)
Supplement: Supplementary file 1 [file DataSheet_1.pdf]

Supplementary Material

Tyrosol facilitates neovascularization by enhancing skeletal muscle cells viability and paracrine function in diabetic hind-limb ischemia mice

Jianqi Zhang, Dyah Ari Nugrahaningrum, Olivia Marcelina, Agnes Dwi Ariyanti, Guixue Wang, Caiping Liu, Shourong Wu*, and Vivi Kasim*

*Email: vivikasim@cqu.edu.cn (V.K.)
shourongwu@cqu.edu.cn (S.W.)

Figure S1

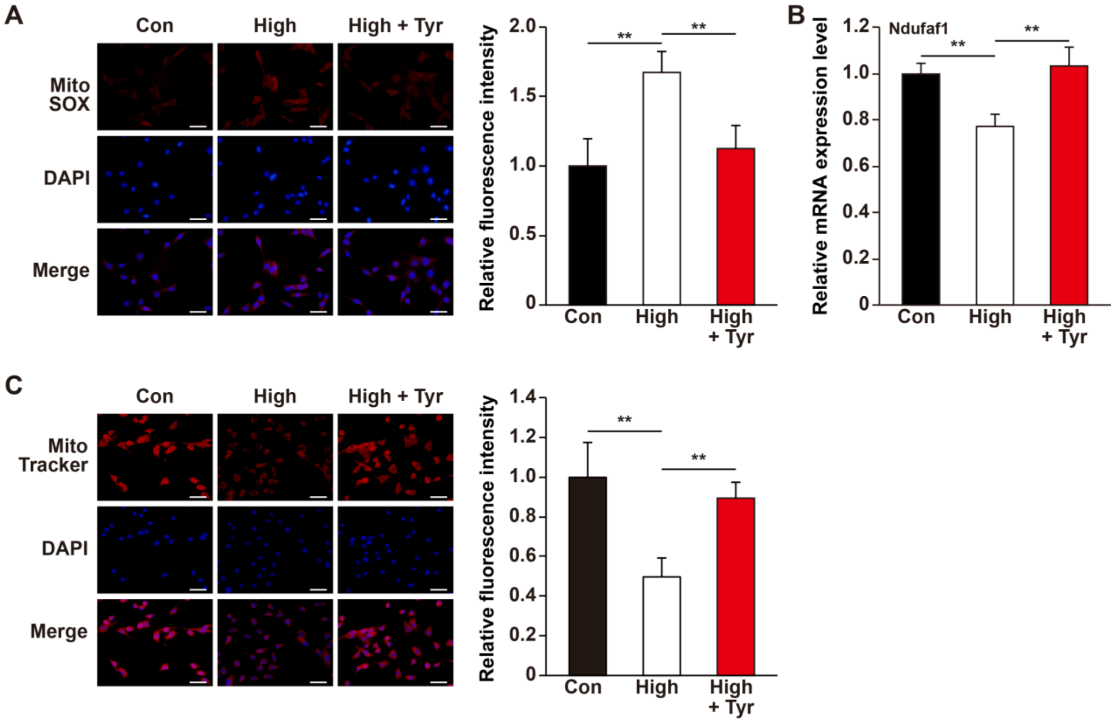

FIGURE S1 | The effect of tyrosol on C2C12 cells mitochondrial oxidative damage. **(A)** Mitochondrial reactive oxygen species (ROS) level in C2C12 cells cultured under hyperglycemia, as examined using MitoSOX staining; left: representative images (scale bars: 100 μm), right: relative fluorescence intensity quantification (n = 6). **(B)** mRNA expression level of Ndufaf1 in C2C12 cells cultured under hyperglycemia, as determined using quantitative reverse transcription PCR (qPCR; n = 3). β-Actin was used for normalization. **(C)** The level of mitochondrial membrane potential of C2C12 cells cultured under hyperglycemia, as examined with MitoTracker Red CMXRos staining; left: representative images (scale bars: 100 μm), right: relative fluorescence intensity quantification (n = 6). Data were shown as relative to that of control, and expressed as mean ± S.D. $^{**}P < 0.01$.

Figure S2

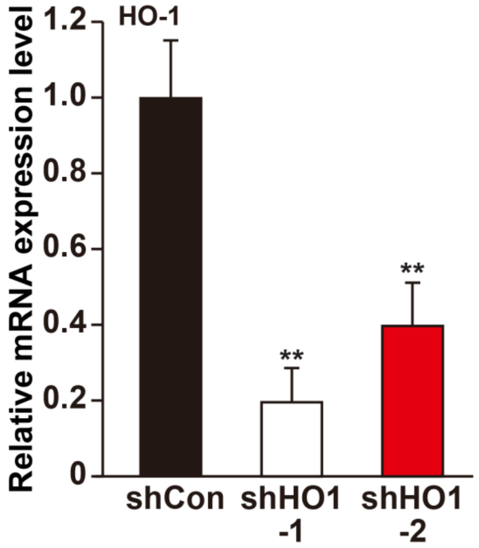

FIGURE S2 | The mRNA expression level of HO-1 in C2C12 cells transfected with shHO-1. HO-1 mRNA expression levels of C2C12 cells transfected with indicated shRNA expression vectors, as determined using quantitative reverse-transcription PCR. β-Actin was used for normalization. Data were shown as relative to that of control, and expressed as mean ± S.D. (n = 3). $^{**}P < 0.01$.

Figure S3

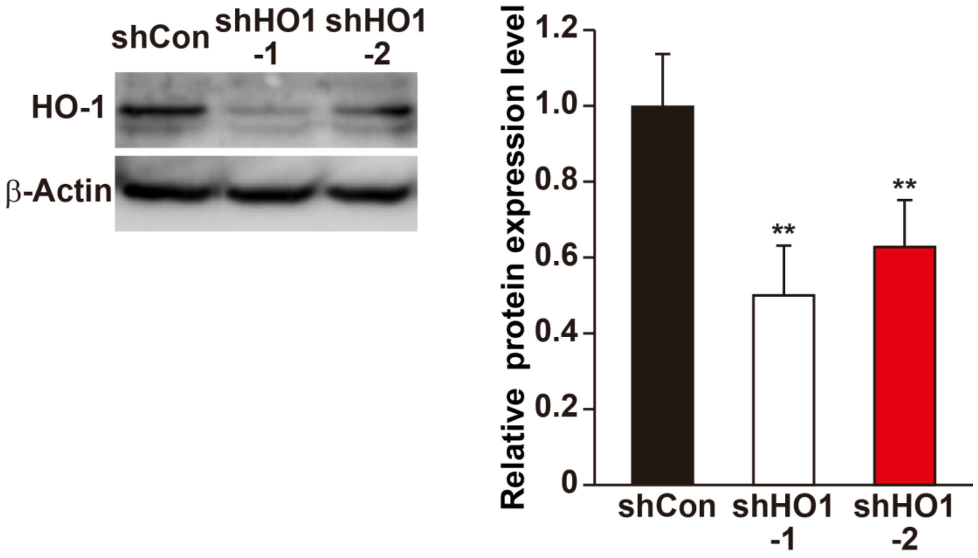

FIGURE S3 | The protein expression level of HO-1 in C2C12 cells transfected with shHO-1. HO-1 protein expression level in C2C12 cells transfected with indicated shRNA expression vectors, as determined using western blotting: representative images (left) and quantification results (right, n = 3) were shown. β-Actin was used as a loading control. Quantification data were shown as relative to that of control, and expressed as mean ± S.D. $^{**}P < 0.01$.

Figure S4

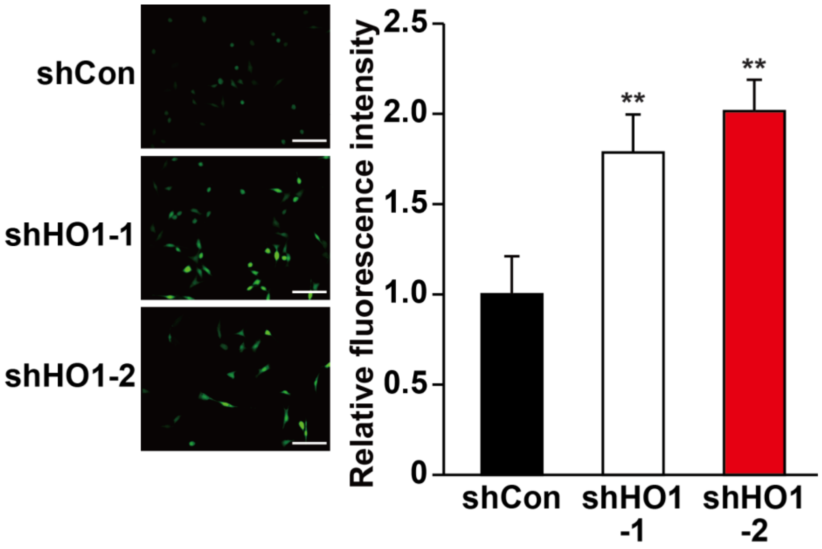

FIGURE S4 | The effect of knocking down *HO-1* on C2C12 cells intracellular ROS level. Intracellular ROS level in *HO-1*-silenced C2C12 cells, as determined using DCFH-DA: representative images (left, scale bars: 200 μm); and quantification of relative fluorescence intensity (right, n = 6) are shown. Data were shown as relative to that of control, and expressed as mean ± S.D. ***P* < 0.01.

Figure S5

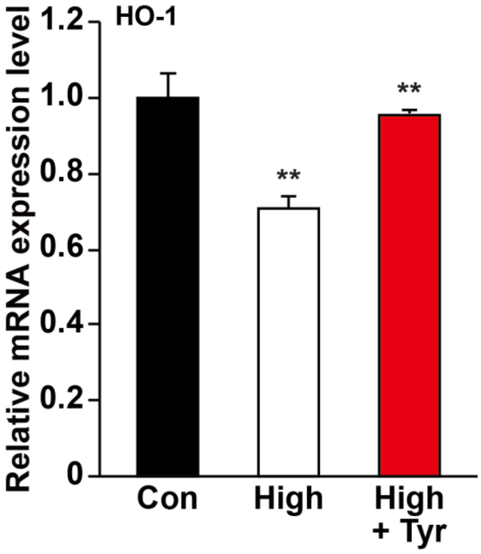

FIGURE S5 | The effect of tyrosol on HO-1 expression level in C2C12 cells cultured under hyperglycemia. The mRNA expression level of HO-1 in C2C12 cells treated with tyrosol, as determined using qPCR. β-Actin was used for normalization. Data were shown as relative to that of control, and expressed as mean ± S.D. (n = 3). ***P* < 0.01.

TABLE S1 | Antibodies and chemicals used for western blotting, immunofluorescence, and phalloidin staining.

Antibody	Product No.	Maker	Experiment	Dilution
anti-VEGF-A	sc-152	Santa Cruz Biotechnology	Western Blotting	1/300
anti-HO-1	sc-10789	Santa Cruz Biotechnology	Western Blotting	1/500
anti-PDGF-BB	sc-7878	Santa Cruz Biotechnology	Western Blotting	1/300
anti-β-Actin	60008-l-Ig	Proteintech	Western blotting	1/20000
anti-PECAM-1	550274	BD Pharmingen	Immunofluorescence	1/100
Monoclonal anti-murine α-SMA Cy3 conjugate	C6198	Sigma-Aldrich	Immunofluorescence	1/50
Alexa Fluor 488 Goat Anti-Rat IgG	A11006	Invitrogen	Immunofluorescence	1/100
Goat Anti-Mouse IgG	ZB2305	ZSGB-BIO	Western Blotting	1/10000
Goat Anti-Rabbit IgG	ZB2301	ZSGB-BIO	Western Blotting	1/10000
Alexa Fluor 555 Phalloidin	A34055	Invitrogen	Phalloidin staining	1/250

TABLE S2 | Blood glucose concentration in diabetic hind-limb ischemia model mice during the experiment.

Control (mmol/l)				Tyrosol (mmol/l)			
**No**	**3w HFD**	**1w after STZ (1d pre-surgery)**	**3w post-surgery**	**No**	**3w HFD**	**1w after STZ (1d pre-surgery)**	**3w post-surgery**
1	8.9	21.6	20.1	1	9.7	20.7	18.7
2	6.3	18.9	18.5	2	7.9	20.9	20.3
3	8.8	20.3	19.2	3	7.4	20.2	17.3
4	8.6	21.9	20.6	4	6.9	22.8	21.5
5	6.9	24.8	24	5	8.2	25.7	24.8
**Mean**	**7.9**	**21.5**	**20.48**	**Mean**	**8.02**	**22.06**	**20.52**
**Stdev**	**1.21**	**2.19**	**2.13**	**Stdev**	**1.06**	**2.26**	**2.87**
***P* value**		**1.97E-6**[#]	**0.477**[$]	***P* value**		**3.83E-08**[#]	**0.374**[$]

HFD: high fat diet; STZ: streptozotocin administration.

[#]*P* value was calculated versus 3 w HFD using one-way ANOVA.

[$]*P* value was calculated versus 1 w after STZ using one-way ANOVA.
